# Natural Changbai mineral water reduces obesity risk through regulating metabolism and gut microbiome in a hyperuricemia male mouse model

**DOI:** 10.3389/fnut.2024.1308882

**Published:** 2024-01-29

**Authors:** Maichao Li, Kai Guo, Yuwei He, Hailong Li, Wenyan Sun, Xuan Yuan, Zhen Liu, Xinde Li, Tony R. Merriman, Changgui Li, Hui Zhang

**Affiliations:** ^1^Shandong Provincial Key Laboratory of Metabolic Diseases and Qingdao Key Laboratory of Gout, The Affiliated Hospital of Qingdao University, Qingdao, China; ^2^Institute of Metabolic Diseases, Qingdao University, Qingdao, China; ^3^Shandong Provincial Clinical Research Center for Immune Diseases and Gout, The Affiliated Hospital of Qingdao University, Qingdao, China; ^4^Medical College, Binhai University, Qingdao, China; ^5^Division of Clinical Immunology and Rheumatology, University of Alabama at Birmingham, Birmingham, AL, United States

**Keywords:** natural mineral water, hyperuricemia mice, body weight, hepatic metabolite profiling, gut microbiota

## Abstract

Access to clean and safe drinking water is essential. This study aimed to evaluate the effect of a kind of small molecular natural mineral water, C-cell mineral water on hyperuricemia male mice metabolism condition. A 13-week drinking water intervention study was conducted in *Uox*-knockout mice (KO). The hepatic metabolite profiling and related genes expression were detected by UPLC-TOF-MS and transcriptomic, and the gut microbiota of KO mice was determined by metagenomics sequencing. Results showed that the body weight of mice fed with C-cell water was remarkably lower than that of control mice on D 77 and D 91. Hepatic metabolite profiling revealed a shift in the pathway of glycine, serine and threonine metabolism, pantothenate and CoA biosynthesis, and biosynthesis of cofactors in KO mice fed with C-cell mineral water. Increased energy metabolism levels were related to increased hepatic expression of genes responsible for coenzyme metabolism and lipid metabolism. Gut microbiota was characterized by increasing activity of beneficial bacteria *Blautia*, and reducing activity of pathobiont bacteria *Parasutterella*. These genera have been reported to be associated with obesity. Small molecular mineral-rich natural water ingestion regulates metabolism and gut microbiota, protecting against obesity induced by hyperuricemia through mediating a microbiota-liver axis.

## 1 Introduction

Access to clean and safe drinking water is a health and social issue given that exposure to poor quality water is harmful to health (1). C-cell mineral water is from the Changbai Mountain area in the eastern part of Jilin Province, China, which is abundant in mineral water resources. The mineral water from Changbai Mountain area is rich in metasilicic acid (2, 3), which is an existing form of silicon in water. Metasilicic acid is mainly derived from the dissolution of sodium-potassium aluminosilicate minerals, polytype variant minerals containing SiO_2_ and fluorine, and clay minerals (4). The water is naturally weak alkaline with pH value 8.5 ± 0.5, containing kinds of minerals and trace elements, such as lithium, strontium, zinc, and selenium, with the levels of all harmful components and elements much lower than the limits set in the National Food Safety Standard Drinking Natural Mineral Water GB8537-2018 standards. In addition, C-cell mineral water is a kind of water with small molecular clusters. The water-hydrogen bond is opened to form a single water molecule through a cutting reactor with nano-scale cutting materials (invention patent number: ZL201410440053.5). As the polarity of water molecules attracts each other and converges into clusters, the polar water molecules are affected by the magnetic field in the process of passing through the strong magnetic field through the long range magnetic induction of the reforming reactor (ZL201310384523.6). According to the right-hand rule, the water molecules obtain the motion-generation electromotive force when cutting the magnetic field lines. Under the action of kinetogenic electromotive force, polar water molecules are arranged in an orderly manner, which affects the vibration behavior of water molecules, forming directional vibration and forming small molecular clusters of water with stable structure. Finally, large particulate matter, organic matter, some heavy metal ions and bacteria were removed through multi-medium filtration system including quartz sand, activated carbon filter layer, and ultraviolet sterilization and ozone sterilization. However, little research has been done on the role of the effect on health of C-cell mineral water.

Energy metabolism is dependent on the existence of various micronutrients, which act as precursors, coenzymes, or essential components at each cell stage. Most enzymes are regulated by organic cofactors and coenzymes, which function as intermediate carrier of functional groups or electrons that are transferred during the metabolic reaction (5). Coenzymes and cofactors play an integral role in many cellular metabolic reactions including lipid metabolism and amino acid metabolism. For example, coenzyme A is involved in hundreds of different anabolic and catabolic reactions, including those responsible for lipid and bile acid metabolism (6). The role of minerals and trace elements supplemented by natural mineral water as cofactors in promoting metabolism needs to be clarified and studied.

Body weight regulation is dependent on a homeostatic system that affects the balance between energy intake and energy expenditure. A growing body of evidence has established that the intestinal microbiota can impact energy homeostasis and body weight (7). It has also been reported that the changes in intestinal microbiota in response to diet can alter energy balance and lead to metabolic disorders and obesity by modulating different pathways involved in coenzyme biosynthesis and fatty acid metabolism (8). However, it is necessary to clarify the molecular mechanisms drivened by diet–microbiota–host interactions, especially by drinking water-microbiota.

Hyperuricemia and obesity have a synergistic association (9). Increased urate levels associate with obesity and type 2 diabetes, and BMI partially mediates the association of urate with risk of diabetes (10). Thus, we used a spontaneous hyperuricemia male mouse model with *Uox* gene (encoding urate oxidase) deficiency to explore the effect of C-cell mineral water on energy metabolism in this study. Analysis included body weight, cecal bacterial composition and diversity, metabolite profiling and gene expression in *Uox*-knockout mice (KO) were performed. We will discuss the research focusing on the benefit to metabolism of drinking mineral water, trace elements supplemented by natural mineral water as cofactor promoting lipid metabolism, and gut microbiota affecting body weight and energy homeostasis, in order to provide support for the hypothesis of drinking natural mineral water on reducing the risk of metabolic disease.

## 2 Materials and methods

### 2.1 Processing of C-cell mineral water

Physico-chemical parameters of analyzed waters were measured using conventional methods. Ion content (Na^+^, K^+^, Mg^2+^, Ca^2+^ mg/L) was assessed by the reference method in China (GB/T 8538-2008) at the SGS-CSTC standards technical services Co., Ltd. All ^17^O-NMR experiments were conducted on a superconductor spectrometer (Bruker, Advance 400 MHz, Bremen, Germany). The pH was determined as an average of six repeated measurements by the universal method using the pH meter (Mettler Toledo, OH, United States).

Processing of waters was performed using procedure with multi-media filtration device with activated carbon filter (Jilin tasly mineral spring beverage co. LTD., Jilin, China). After that, the large water molecule clusters were cut into small water molecule clusters after going through the reactor filled with nano-scale cutting material. Finally, the water molecules were arranged in order to form a stable structure of small molecular clusters by reforming the reactor (11).

### 2.2 Instruments and reagents

Mass Spectrometer (QTOF/MS-6550, Aglient), Ultra-High Performance Liquid Chromatograph (UPLC-1290, Aglient), Vortex mixer (MIX-200, Jingxin, Shanghai), centrifuge (5427R, Eibend, Germany), acetonitrile and methanol were bought from Merck (Darmstadt, Germany). Formic acid and 2-chlorophenylalanine were from Thermo Fisher. All chemicals were of chromatographic grade.

### 2.3 Animals and experimental design

Animal experiments were performed in compliance with the principles of laboratory animal care and with approval by the Animal Ethical and Welfare Committee of Affiliated Hospital of Qingdao University. Male C57BL/6 *Uox*-knockout mice (KO) of eight-week-old with average body weight of 20 ± 2 g were initially fed for 14 days in a specific pathogen free level animal house. After acclimation, mice with normal body weight were randomly assigned to 2 groups (6–8 mice per group): control group (KO mice supplemented with ultrapure H_2_O) and treated group (KO mice supplemented with C-cell mineral water). Water was changed every 24 h. Simultaneously, the content from colon of mice was harvested to assess gut microbiota (repeats = 6/group). All mice were euthanized by CO_2_ (30% < VDR/min < 70%). After completion of the experiment, blood samples were drawn from the outer canthus for liver biochemical index analysis. To isolate the liver and gut, the sacrificed mice were dissected. The tissues were frozen in liquid nitrogen until analysis. The study was performed according to animal testing guidelines (the National Research Council’s Guide for the Care and Use of Laboratory Animals), and was approved by the Qingdao University Institutional Review Board (No: QDU-AEC-2022426).

### 2.4 Determination of body weight and biochemical index

The water intake of all mice was recorded daily. The body weight (g) of all mice was recorded during the experimental stage (every 14 days) and after completion of the experiment. Mice were fasted overnight before biochemical blood tests. Biochemical measurements were assessed using standard laboratory methods with a biochemical analyzer for small animals (Mindray, BS-240VET, Shenzhen, China). Blood was withdrawn from the outer canthus of euthanasia mice and placed in a sterile isolation tube for immediate detection of biochemical indicators including serum urate (SU), glucose (GLU), alanine transaminase (ALT), aspartate transaminase (AST), triglycerides (TG), urea nitrogen (BUN), total cholesterol (TC), and serum creatinine (SCR).

### 2.5 Liver metabolomic analysis

To profile the holistic alterations in the metabolomic fingerprints, 25 mg livers tissues from each mouse were extracted, using the method from Wu et al., but with some differences (12). The extracts were performed with UPLC-MS by Metware Biotechnology Co., Ltd. (Wuhan, China). Detailed methods for extraction, identification, and data analysis are provided in the Supplementary Text S1. To ensure both accuracy and precision, quality control measures were undertaken.

### 2.6 Metagenomic sequencing and gut microbiota analysis

Total genomic DNA was extracted from gut content (10 mg) of mice using the E.Z.N.A.^®^ Soil DNA Kit (Omega Bio-tek, GA, United States). The amplified DNA was subjected to library preparation (Agilent 2100/Q-PCR) and then sequenced on an Illumina NovaSeq platform (PE 150; Majorbio Biotechnology Co., Ltd., Shanghai, China). Pre-processing of sequencing data was conducted to retrieve clean data. The details are described in the Supplementary material. Data analysis was conducted using the online Majorbio cloud platform^1^ (13).

### 2.7 Transcriptomic analysis

Total RNA from liver was sequenced using the second-generation high-throughput sequencing technique (Metware, Wuhan, China). More details of library preparation, sequencing and differential analysis are described in the Supplementary material.

### 2.8 Statistical analysis

One-way and two-way ANOVA followed by Tukey *post-hoc* testing was conducted to assess statistical significance (*p* < 0.05). The analysis method of microbial and metabolite data are presented in the Supplementary material.

### 2.9 Data availability statement

Raw sequence data of metagenomic sequencing were deposited into the NCBI Short Read Archive database (Accession Number: PRJNA883209). Raw sequence data of transcriptomic sequencing were deposited into the NCBI Short Read Archive database (Accession Number: PRJNA903546).

## 3 Results

### 3.1 Characteristics of the C-cell mineral water

**Figure 1:**
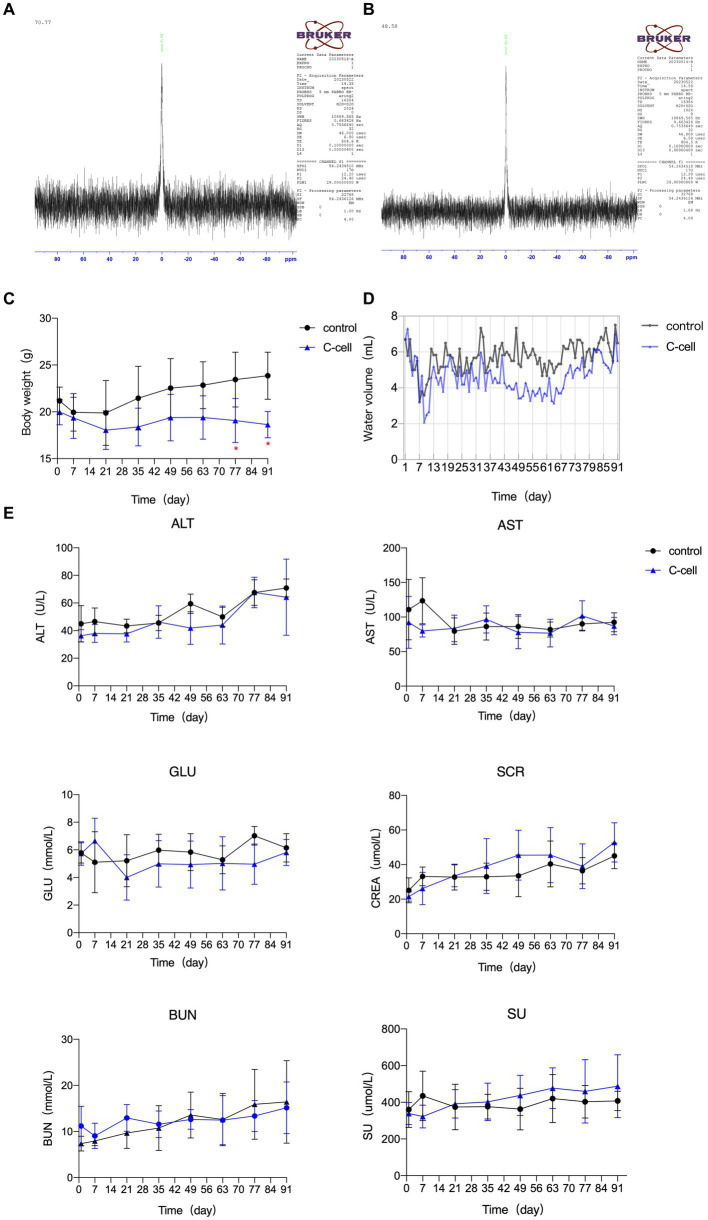
^17^O-NMR half peak width of general water **(A)**. ^17^O-NMR half peak width of small molecule C-cell mineral water **(B)**. Body characteristics and serum biochemical indexes in KO mice fed C-cell mineral H_2_O or ultrapure H_2_O for 91 days. Water intake **(C)**, body characteristics **(D)**, serum biochemical indexes **(E)**. Data are expressed as mean ± SEM or ± SD. *n* = 6–8. *p < 0.05; ∗∗*p* < 0.01. Control mice fed ultrapure H_2_O; C-cell mice fed C-cell mineral H_2_O.

The characteristics of the C-cell mineral water are displayed in Table 1. ^17^O-NMR half peak width is 70.77 MHz for general water, and 48.58 MHz for the small molecule C-cell mineral water (Figures 1A,B).

**Table 1:** Chemical and physical properties of the C-cell mineral water.

Index	Value
pH	8.5 ± 0.5
Conductivity (μS/cm)	150–300
Bromine (mg/L)	<0.01
Potassium (mg/L)	0.1–6.0
Sodium (mg/L)	6.0–70.0
Calcium (mg/L)	0.1–15.0
Magnesium (mg/L)	0.5–11.0
Metasilicic acid Calcium Sodium (mg/L)	30.0–55.0
^17^O-NMR half peak width (MHz)	48.58
Total number of colonies (CFU/g)	<10

### 3.2 Analysis of body characteristics and serum biochemical indexes

The body weight of control mice, but not C-cell-fed mice, gradually increased. The body weight of mice fed with C-cell water was remarkably lower than that of control mice on D 77 and D 91 (Figure 1C). The 24-h water intake of each mouse in the C-cell water group was lower, but not significantly, than that in control group (Figure 1D). The GLU, AST, ALT, SCR, SU, and BUN levels in serum were not apparently different between the C-cell-fed and control groups (Figure 1E).

### 3.3 Drinking water changed metabolic pathway in *Uox-*KO mice

**Figure 2:**
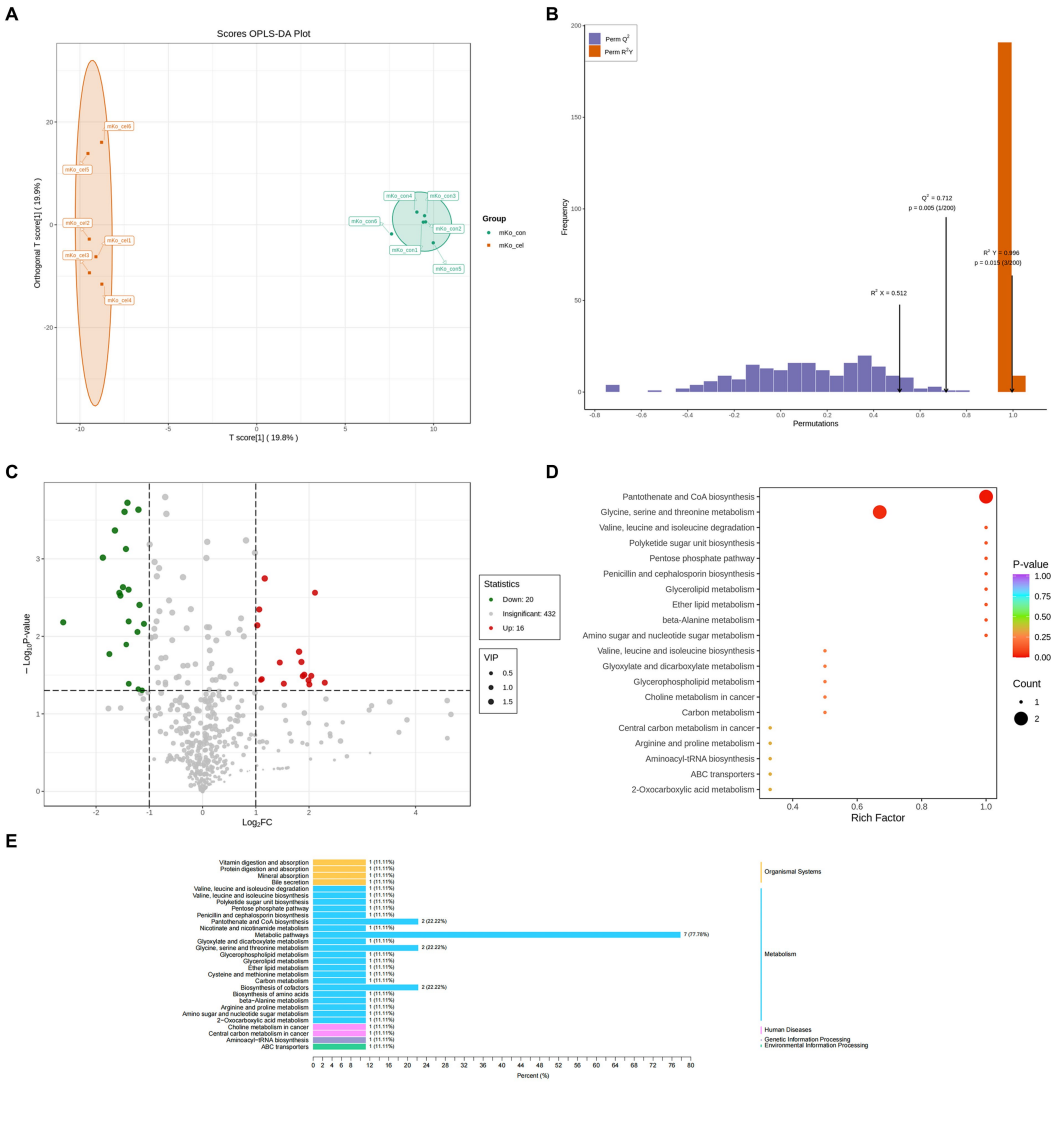
Drinking C-cell water alters mice liver metabolic profiles. Scatter plot of the OPLS-DA scores of liver metabolites in the control and C-cell mineral water groups with positive ionization modes of UPLC-MS in knockout (KO) mice **(A,B)**. The volcano plot demonstrated perturbed metabolites **(C)** in KO mice in the C-cell mineral water group compared with controls (red, up regulated; green, down regulated). The data set was screened according to the variable importance for projection (VIP) values ≥1, and the fold-change values ≥1 and ≥2 for WT mice and KO mice, respectively; or fold change ≤0.5, *p*-value < 0.05. KEGG pathway analysis of differential metabolites in KO mice **(D,E)** with the C-cell mineral water group compared with controls. Enrichment factor is the ratio of the number of differentially expressed metabolites (DEMs) in the corresponding pathway to the total number of metabolites identified and annotated in the pathway. The greater the enrichment degree, the larger the value. *p*-value is the hypergeometric test *p*-value: the closer it is to 0, the more significant the enrichment is. The size of the point is related to DEMs enriched in the corresponding pathway. *N* = 6 in each group.

Liver specimens collected from the control and treated groups were detected by UPLC-TOF-MS. Peptides, cofactors, vitamins and lipids were the dominant types of metabolites (Supplementary Table S1). The metabolomic difference between groups was evaluated by principal components analysis (PCA) (Supplementary Figure S1). The score plot of the Orthogonal Partial Least Squares-Discriminant Analysis (OPLS-DA) model corresponding to the PCA model is also shown. An obvious separation for between-group comparison was found in the score plot of the OPLS-DA model (*R*^2^ X = 0.512, *R*^2^ Y = 0.996 and *Q*^2^ = 0.712, *p* < 0.05) (Figures 2A,B). These findings demonstrated that treatment changed metabolite levels in the liver. A volcano plot showed that 36 differential metabolites were identified between the two groups at *p* < 0.05 and VIP > 1 (Figure 2C). Sixteen of the 36 metabolites were increased in the C-cell water group, and twenty were decreased compared to the control group (Supplementary Table S1). The differential metabolic pathway was involved in the metabolism of glycine, serine and threonine metabolism (22.2%), pantothenate and CoA biosynthesis (22.2%) and biosynthesis of cofactors (22.2%) (Figures 2D,E).

### 3.4 Drinking water alters the composition and function of intestinal microbiota in KO mice

**Figure 3:**
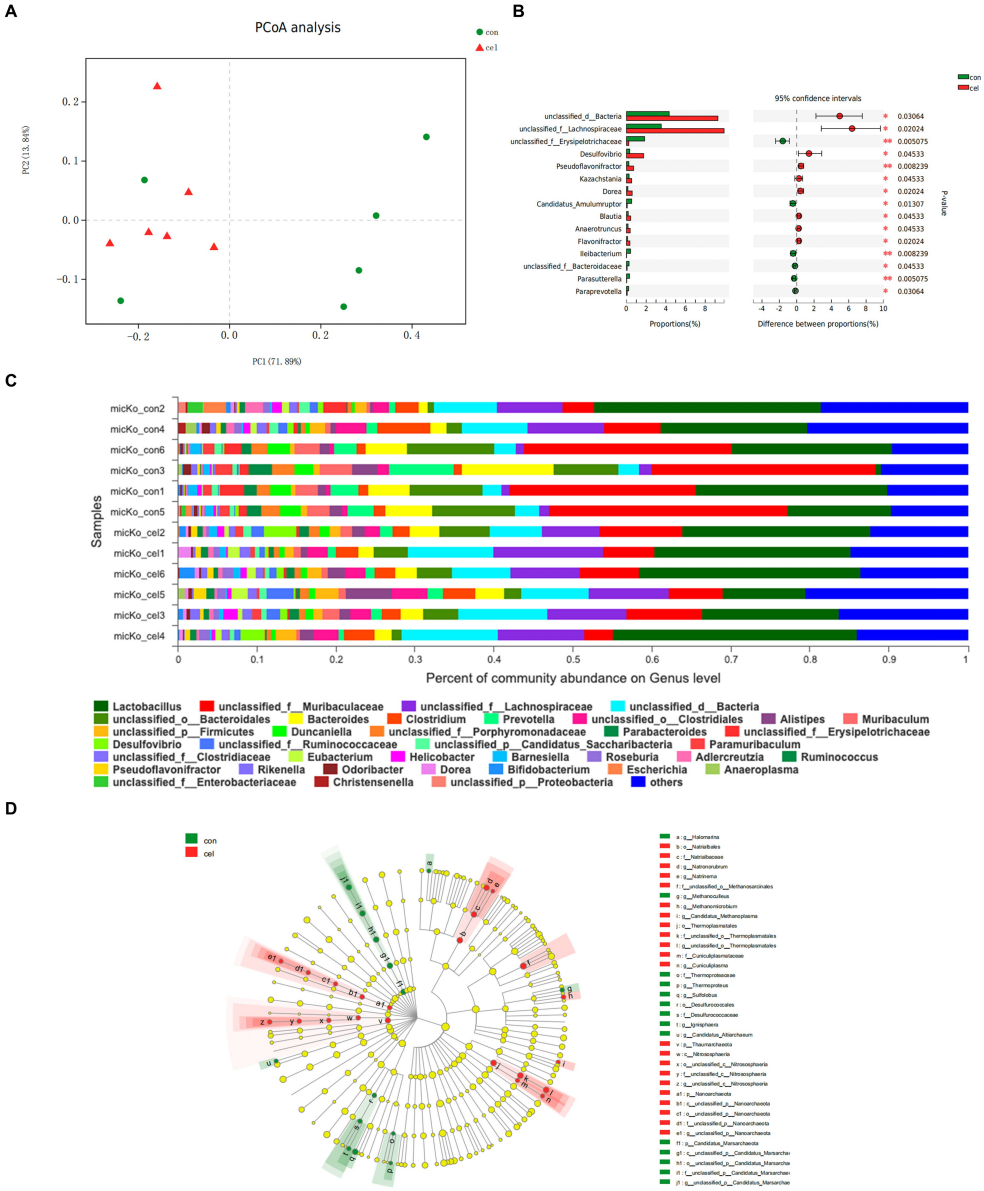
Effects of C-cell water on the gut microbiome composition. **(A)** OPLS-DA analysis of operational taxonomic units (OTUs) abundance segregating controls (green) from C-cell water group (red). **(B)** The significance of the difference between C-cell water and control group based on Wilcoxon rank-sum test bar plot on genus level. *p < 0.05; and **p < 0.01 for the comparison. **(C)** The microbiome composition at the genus level. **(D)** Cladogram analysis effect size analysis from phylum to genus of differential gut microbiota between the C-cell water and control groups. The circle radiating from inside to outside indicates the classification level from phylum to genus. The diameter of the small circle is positively related to the relative abundance. *N* = 6 in each group.

To directly investigate the impact of drinking C-cell water on KO mice gut microbiota, metagenome sequencing was conducted. A total of 554 Operational Taxonomic Units (OTUs) including 5 domains, 14 kingdoms, 200 phyla, 341 classes, 600 orders, 1,044 families, 2,978 genera, and 13,089 species were observed. We carried out principal coordinate analysis (PCoA) of unweighted UniFrac distances between groups to determine the effects of different water on the microbiota. An obvious separation between the communities was found at the first principal coordinate (x axis), which could explain 71.89% of the variance. The second principal coordinate (y axis) accounted for 13.84% of the variance and separated the communities (Figure 3A). Analysis at the genus level revealed that mice fed with C-cell water were associated with decreases in *Muribaculaceae* and *Bacteroidetes*, while an increase in *Lachnospiraceae* (Figure 3C). To assess the changes in the microbiota of each group, a linear discriminant analysis integrated with effect size (LEfSe) analysis was conducted to identify the dominant microorganisms in each group. This result indicated that genera *Natronorubrum, Natrinema, Methanomicrobium, Candidatus_Methanoplasma, Cuniculiplasma* and quite a few of other genera were significantly altered compared with fed with C-cell water (Figure 3D). A comparative analysis of the two groups found that genera *Lachnospiraceae, Desulfovibrio, Pseudoflavonifractor, Kazachstania, Dorea, Blautia, Anaerotruncus* and *Flavonifractor* were significantly increased in the C-cell water group (Figure 3B).

**Figure 4:**
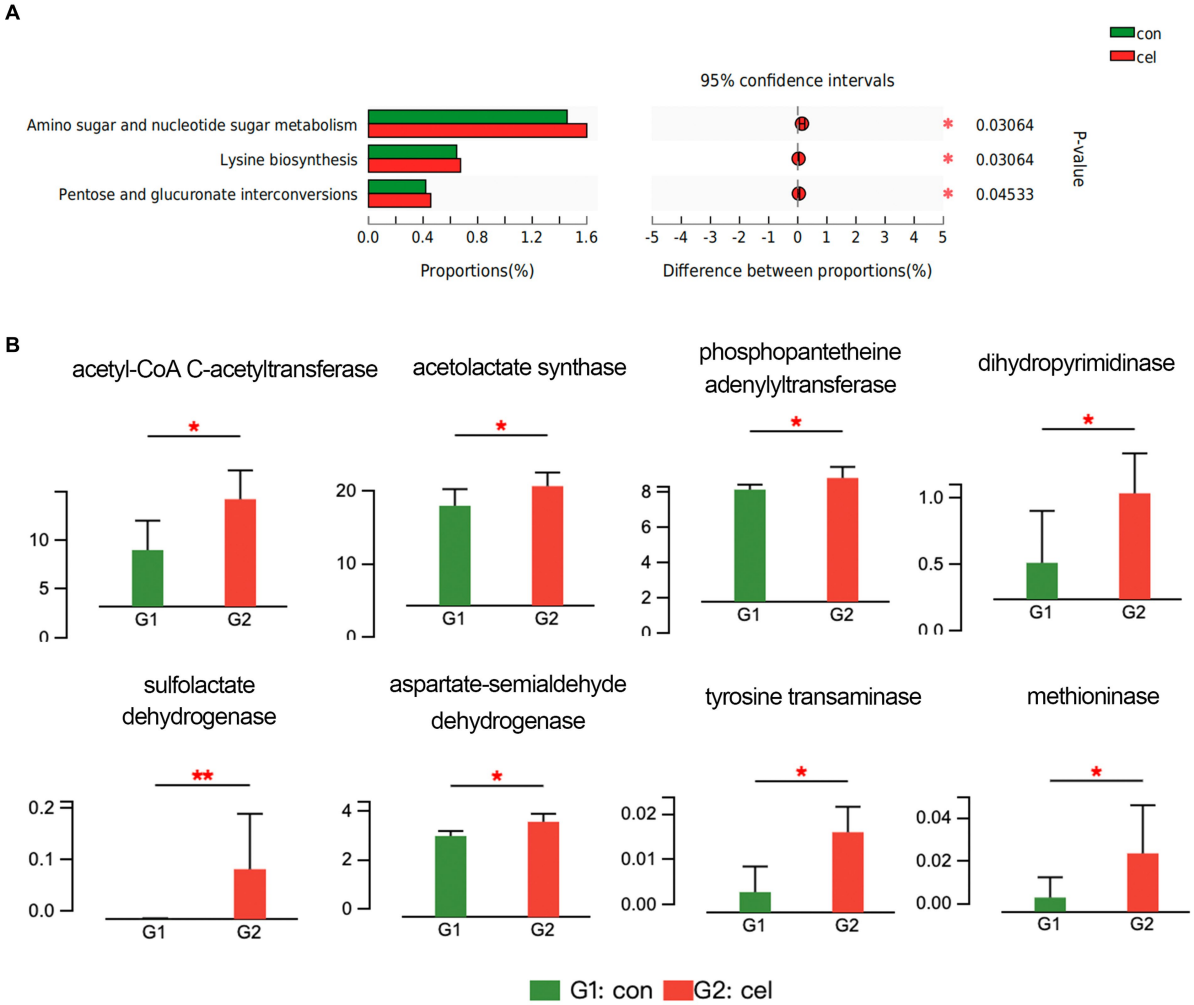
Effects of C-cell water on the gut microbiome composition. **(A)** KEGG pathway analysis of the intestinal microbiome between C-cell water and control group. **(B)** The different analysis of enzyme related to key metabolism pathways. *p < 0.05; and **p < 0.01 for the comparison.

Based on the KEGG database, upregulated pathways included nucleotide sugar and amino sugar metabolism, pentose and glucuronate interconversions, and lysine biosynthesis pathways in the C-cell water group (Figure 4A). In addition, the potential functions of intestinal microbiota could be attributed to the changes in enzyme activities. According to the enzyme nomenclature database, there were 120 differentially expressed enzymes between two groups. Among these enzymes, acetyl-CoA C-acetyltransferase (2.3.1.9) in the C-cell water group was enhanced compared to control in the fatty acid degradation pathway (ko00071) (Figure 4B). Moreover, C-cell water treatment increased the expression of acetolactate synthase (2.2.1.6), phosphopantetheine adenylyltransferase (2.7.1.24) and dihydropyrimidinase (3.5.2.2) in the pantothenate and CoA biosynthesis pathway (ko00770). An increased expression of sulfolactate dehydrogenase (1.1.1.338), aspartate-semialdehyde dehydrogenase (1.2.1.11), tyrosine transaminase (2.6.1.5), and methioninase (4.4.1.11) was involved in the cysteine and methionine metabolism pathway (ko00270) (Figure 4B).

### 3.5 Drinking water alters the expression of genes associated with energy metabolism

For gene expression analysis, the liver tissues with four duplicates were collected from two groups. A total of 233 differentially expressed genes (DEGs) were identified. Among which, 85 were up-and 148 down-regulated (Supplementary Figure S1). Among these, six genes related to coenzymes and energy metabolism are listed in Table 2.

**Table 2:** Upregulated expression of genes in the water group compared to control.

Gene name	Gene description	log2FoldChange	*p*-value
COQ10b	Coenzyme Q10b	2.1378	4.6798E-25
POR	P450 (cytochrome) oxidoreductase	1.5683	4.7311E-5
ACOT3	Acyl-CoA thioesterase 3	3.3280	2.8370E-4
PFKFb3	6-phosphofructo-2-kinase	1.7660	7.2420E-4
NAMPT	Nicotinamide phosphoribosyltransferase	1.0763	6.5751E-3
PPARα	Peroxisome proliferator activated receptor alpha	1.5409	7.6712E-3

## 4 Discussion

Previous studies have reported the beneficial effects of drinking mineral water on human health (14–17). However, whether drinking natural mineral water can prevent obesity caused by metabolic disease such as hyperuricemia has not been reported, particularly from the perspective of metabolic levels and gut microbiota. Our lab previously established a spontaneous hyperuricemia mouse model with *Uox* gene (encoding urate oxidase) deficiency using the transcription activator-like effect or nuclease (TALEN) technique (18). Here, multi-omics approaches were performed, and the results showed that a kind of small molecule mineral water can regulate energy metabolism in *Uox*-knockout mice, and further may protect against the obesity induced by hyperuricemia.

### 4.1 Drinking mineral water is beneficial to metabolism

Urban water supply networks are vulnerable to accidental/intentional biological and chemical pollution, which pose a threat to human health. Recently, the incidents of drinking-water pollution can happen regularly, thereby endangering social security and stability (19). The safety of drinking-water is important. The significant increase in mineral water sales as a daily drink can reflect the continuous confidence in spa treatment. Minerals elements and active molecules/ions present in mineral waters (and their pH) are important to counterbalance inadequate intake from other sources and consequent metabolic dysfunction and an increase in diet-induced acid-load in metabolic syndrome (20). Previous research has shown that Avene thermal spring water exhibits immunomodulatory potential (21). Studies on the hydromineral therapy effects of Evian mineral water have been carried out over >50 years (22). Research on the effect of mineral water demonstrated positive effect on regulating glucose level in T2DM and the reduction of risk of metabolic syndrome (15, 23, 24). Additionally, C-cell water is rich in metasilic acid, which is a form of silicon present in the body. It has been shown that silicon thermal water corrects lipid metabolism in children with dysmethabolic nephropathy (25). However, little research has been done on small molecule water. This study confirmed that C-cell water can regulate energy metabolism. Although we cannot draw a definite conclusion on whether the key effector components are small molecules or minerals, or both. Further studies are warranted for determining the biochemical mechanism of small molecule water.

### 4.2 Trace elements supplemented by natural mineral water as cofactor promoting lipid metabolism

Natural mineral water contains a large amount of trace elements. Essential trace elements play a crucial role in the maintenance of health, as they are responsible for numerous metabolic pathways (26) through influencing the structures of complex carbohydrates, enzymes, and proteins. Studies reported that deficiency of trace and mineral elements is closely associated with the pathogenesis of systemic disorders and metabolic diseases (27). In our study, hepatic metabolite profiling indicated that the most enriched differential metabolic pathways included pantothenate and CoA biosynthesis and biosynthesis of cofactors. The transcriptomic level of coenzyme Q10 (CoQ10) and nicotinamide phosphoribosyl transferase (NAMPT) relating to coenzyme metabolism were also significantly increased. CoQ10 can serve as a lipid-soluble antioxidant (as a cofactor of the enzyme dihydroorate dehydrogenase), which play essential roles in lysosomal, pyrimidine and fatty acid metabolism (28). CoQ10 supplementation has been shown to improve the level of lipid metabolism and ameliorates obesity (29). NAMPT is associated with the pathogenesis of obesity, type 2 diabetes and nonalcoholic fatty liver disease (NAFLD), by affecting lipid and glucose metabolism (30). CoA is involved in more than hundreds of different anabolic and catabolic reactions, including those responsible for bile acid and lipid metabolism (6), which could support the findings of this study.

The increased expression of PPAR-α and POR mRNA in our study indicated that long-term intake of mineral water could activate pathways related to lipid metabolism. The peroxisome proliferator-activated receptors (PPARs) are important regulators bridging trace elements and metabolic homeostasis (31). PPARs are closely associated with different metabolic processes, including adiposity (32). Research also shows that activating PPAR-α in the liver can ameliorate obesity-induced metabolic abnormalities (33). PPARs also sensitively respond to changes in trace elements. For instance, zinc has been shown to be closely associated with the DNA-binding activity of PPAR-α (34). P450 oxidoreductase (POR) is an electron donor for all microsomal P450 enzymes, which can be related to lipid metabolism (35). Systemic effects of POR knockdown on global protein expression were evidenced by the downregulation of various metabolic pathways such as biological oxidation reactions and lipid metabolism in a human liver cell model (36).

### 4.3 Gut microbiota affects body weight and energy homeostasis

We assessed the changes and effects of the intestinal flora using the metagenome sequencing approach and clarified whether the changes were associated with weight. Recent characterizations of the mineral water microbiome indicated that it had a significant impact on bacteria (37). It has been reported that bicarbonate-rich mineral water changes the fecal gut microbiota composition in healthy subjects. Mineral water consumption vs. tap water consumption reduced the indexes of glycemic control, altered the blood metabolome, and increased the fecal composition of lean-inducible bacteria after intake (38). Here, the composition of the microbiota was changed after C-cell water intake, as evidenced by the increased abundance of beneficial bacteria, such as *Blautia* relating to obesity, which has been identified as beneficial bacteria associating with improvements in glucose and lipid homeostasis in an open label, randomized, multicenter clinical trial (39). A cross-sectional study of Japanese adults showed that the *Blautia* genus was inversely related to type 2 diabetes mellitus and obesity (40). In this study, we screened a decrease in *Parasutterella* sp. at the genus level, which is reported to be a pathobiont bacteria associated with metabolic abnormalities in rodents and could respond to a high-fat diet intervention in obesity-prone mice (41). Previous research has confirmed that *Parasutterella* is positively associated with BMI and type 2 diabetes in a translational human study (42). In addition, a magnetic resonance imaging study demonstrated that *Parasutterella* was related to hypothalamic inflammation in obese subjects, which is believed to interfere with satiety and appetite regulation, thereby leading to the occurrence of obesity (42). Our study also found an increase of *Pseudoflavonifractor* genus in the C-cell water group, which is consistent with a cross-sectional study that reported that obese patients who succeeded in weight loss had at baseline a microbiota enriched in *Pseudoflavonifractor*, compared to patients who were less successful in weight reduction (43). Furthermore, the potential impact of intestinal microbiota could also result from changes in enzyme activities. In the present study, enzymes related to fatty acid degradation, pantothenate and CoA biosynthesis, and amino acid metabolism pathway were activated in C-cell water intake mice. This phenomenon indicates that C-cell water may affect the host’s metabolism by modulating the metabolic pathway of gut microbiota (44).

## 5 Conclusion

This study showed that the body weight of mice fed with C-cell water was remarkably lower than that of control mice on D 77 and D 91. Hepatic metabolite profiling revealed a shift in the pathway of glycine, serine and threonine metabolism, pantothenate and CoA biosynthesis, and biosynthesis of cofactors in KO mice fed with C-cell mineral water. Increased energy metabolism levels were related to increased hepatic expression of genes responsible for coenzyme metabolism and lipid metabolism. Gut microbiota was characterized by increasing activity of beneficial bacteria *Blautia*, and reducing activity of pathobiont bacteria *Parasutterella*. These genera have been reported to be associated with obesity. In addition, the potential functions of intestinal microbiota could be attributed to the changes in enzyme activities. Based on the enzyme nomenclature database, C-cell water enhanced intestinal microbiota enzyme activities related to fatty acid degradation pathway (ko00071), pantothenate and CoA biosynthesis pathway (ko00770), and cysteine and methionine metabolism pathway (ko00270). Small molecular mineral-rich natural water ingestion regulates metabolism and gut microbiota, protecting against obesity induced by hyperuricemia through mediating a microbiota-liver axis.

The findings provide support for the hypothesis of small molecular mineral-rich natural water ingestion regulating metabolism and gut microbiota of mice, protecting against obesity induced by hyperuricemia through mediating a microbiota-liver axis.

## Data availability statement

The original contributions presented in the study are publicly available. This data can be found here: https://www.ncbi.nlm.nih.gov/, BioProject: PRJNA883209 and PRJNA903546.

## Ethics statement

The animal study was approved by Approval document of Ethics Committee Medical College of Qingdao University. The study was conducted in accordance with the local legislation and institutional requirements.

## Author contributions

ML: Methodology, Writing – original draft, Writing – review & editing. KG: Methodology, Writing – original draft, Writing – review & editing. YH: Data curation, Writing – review & editing. HL: Writing – review & editing. WS: Software, Writing – review & editing. XY: Validation, Writing – review & editing. ZL: Methodology, Writing – review & editing. XL: Conceptualization, Writing – review & editing. TM: Writing – review & editing. CL: Funding acquisition, Supervision, Writing – review & editing. HZ: Writing – original draft.

## Footnotes

^1^http://www.majorbio.com
