# Supplementary material for: Natural Changbai mineral water reduces obesity risk through regulating metabolism and gut microbiome in a hyperuricemia male mouse model
Source: Front Nutr. 2024 Jan 29;11:1308882. doi: 10.3389/fnut.2024.1308882 (PMC10859528; doi:10.3389/fnut.2024.1308882)
Supplement: Supplementary file 1 [file Data_Sheet_1.docx]

Text S1 **Non-targeted metabolomics**

**Tissue sample preparation for untargeted metabolome analysis**

After homogenization by a grinder (30 HZ) for 20 s, the sample (20 mg) was added with 400 μL Methanol:Water solution (ratio= 7:3, V/V) containing internal standard, and then shaken at 1500 rpm for 5 min. The samples were placed on ice for 15 min, followed by centrifugation at 12000 rpm for 10 min at 4 °C. Then, 300 μL supernatant was harvested and placed at -20 °C for 30 min. After centrifugation at 12000 rpm for 3 min at 4 °C, 200 μL aliquots of supernatant were subjected to LC-MS analysis.

**UPLC-MS/MS condition for untargeted metabolome analysis**

**UPLC-MS/MS condition for untargeted metabolome analysis**

All samples were analyzed using the LC-MS system. The analytical conditions were as follows: UPLC column, waters ACQUITY UPLC HSS T3 C18 (1.8 µm, 100 mm*2.1 mm); injection volume, 2 μL; flow rate, 0.4 mL/min; column temperature, 40 °C; solvent system, water (0.1% formic acid):acetonitrile (0.1% formic acid); gradient program, 95:5 V/V at 0 min, 10:90 V/V at 11 min, 10:90 V/V at 12 min, 95:5 V/V at 12.1 min, 95:5 V/V at 14 min.

The raw data file acquired by LC-MS was converted into mzML format by ProteoWizard software. Retention time correction, peak alignment and peak extraction were respectively conducted by XCMS program. The “SVR” method was employed to correct the peak areas. The peaks with detection rate lower than 50 % in each group of samples were discarded.

**Metabolite data analysis**

Identificatio of metabolites was facilitated by searching the laboratory’s self-built database, integrated public database, and AI database. Unsupervised PCA was conducted using the function prcomp in R (www.r-project.org). The data were unit variance scaled before unsupervised PCA. The hierarchical cluster analysis (HCA) data of samples and metabolites are displayed as a heatmap with dendrograms, whereas the Pearson correlation coefficient (PCC) of each sample was determined using the cor function in R and expressed as a heatmap. Both PCC and HCA were calculated using the ComplexHeatmap in R. For HCA, the normalized signal intensities of metabolites (unit variance scaling) are displayed as a color spectrum. For two-group analysis, differential metabolic pathway analysis was conducted using the MetaboAnalyst5.0, with an impact value > 0.1. determined by VIP (VIP ≥ 1), P-value (P<0.05, Student’s t test) and absolute Log2FC (|Log2FC| ≥ 1.0). The VIP value was obtained from the OPLS-DA data, which also contains permutation plot and score plot, and was produced using the MetaboAnalystR in R. The data were log transformed (log2) and mean centered prior to OPLS-DA. A permutation test (200 permutations) was carried out to avoid overfitting. The identified metabolites were annotated based on the KEGG compound database (<http://www.kegg.jp/kegg/compound/>), and subsequently mapped to KEGG Pathway database (<http://www.kegg.jp/kegg/pathway.html>). The significantly enriched pathways were determined using a hypergeometric test P-value for a given list of metabolites.

**Text S2 Metagenomic sequencing**

The purity and yield of DNA extracts were assessed by NanoDrop2000 and TBS-380, respectively. The extracted DNA was fragmented to a mean size of 400 bp via Covaris M220 (Gene Company Limited, China) for paired-end library construction. The paired-end library was established with NEXTflexTM Rapid DNA-Seq (Bioo Scientific, TX, USA). Paired-end sequencing was conducted at Majorbio Bio-Pharm Technology (Shanghai, China) on Illumina NovaSeq/Hiseq Xten (Illumina Inc., CA, USA) using the NovaSeq Reagent Kits/HiSeq X Reagent Kits in accordance with the kit’s protocols (www.illumina.com). The clean reads were obtained by removing low-quality reads (reads with N bases, a minimum quality threshold of 20 and a minimum length threshold of 50 bp) with fastp12 (https://github.com/OpenGene/fastp, v0.20.0) on the freely available online Majorbio Cloud Platform (cloud.majorbio.com). To detect and eliminate the human host originated reads, the clean reads were mapped to the mouse reference genome (mus_musculus) using the BWA13 (http://bio-bwa.sourceforge.net, v0.7.9a). These reads were subsequently assembled to contigs using the MEGAHIT14 (parameter setting: step=10, kmer_max=97, kmer_min=47) (<https://github.com/voutcn/megahit>, v1.1.2) via succinct de Bruijn graphs. Contigs (≥300 bp) were chosen for final assembly. Sequence data generated from this study were deposited into the NCBI Short Read Archive database (Accession Number: PRJNA883209).

**Metagenomic data analysis**

Open reading frames (ORFs) in contigs were determined by MetaGene15 (http://metagene.cb.k.u-tokyo.ac.jp/). The predicted ORFs (≥100 bp) were obtained and translated into amino acid sequences using the NCBI translation table ([http://www.ncbi.nlm.nih.gov/Taxonomy/taxonomyhome.html/index.cgi?chapter=tgencodes#SG1](http://www.ncbi.nlm.nih.gov/Taxonomy/taxonomyhome.html/index.cgi?chapter=tgencodes#SG1)). A non-redundant gene catalog was constructed using CD-HIT16 (<http://www.bioinformatics.org/cd-hit/>, v4.6.1) with 90% coverage and 90% sequence identity. After quality control, the reads were mapped to the non-redundant gene catalog with 95% identity using the SOAPaligner 17 (http://soap.genomics.org.cn/, v2.21), and gene abundance in all samples was assessed. [The sequences](http://en.wikipedia.org/wiki/Representative_sequences) of the non-redundant gene catalog were annotated based on the NCBI NR database using the blastp in DIAMOND v0.9.19 (e-value cutoff = 1e-5). Diamond18 (http://www.diamondsearch.org/index.php, v0.8.35) was employed for taxonomic annotation. Cluster of orthologous groups of proteins (COGs) annotation was conducted for the [representative sequences](http://en.wikipedia.org/wiki/Representative_sequences) using the Diamond18 (http://www.diamondsearch.org/index.php, v0.8.35) against the eggNOG database v4.5.1 (e-value cutoff = 1e-5). The KEGG annotation was performed using the Diamond18 (http://www.diamondsearch.org/index.php, v0.8.35) against the KEGG database (http://www.genome.jp/keeg/, v94.2, e-value cutoff = 1e-5).

**Text S3 Transcriptomic analysis**

Poly-T oligo attached magnetic beads were employed to purify mRNA from total RNA. Fragmentation was performed with divalent cations in NEBNext First Strand Synthesis Reaction Buffer (5X) at elevated temperature. The random hexamer primer and M-MuLV Reverse Transcriptase (RNase H-) were used for first-strand cDNA synthesis, while DNA Polymerase I and RNase H were applied for second-strand cDNA synthesis. Remaining overhangs were converted into blunt ends through polymerase/exonuclease activities. After adenylation of 3’-ends of DNA fragments, NEBNext Adaptors with hairpin loop structure were ligated for hybridization. To select cDNA fragments of 250~300 bp, the library fragment was purified using the AMPure XP system (Beckman Coulter, Beverly, USA). Subsequently, 3 μl USER Enzyme (NEB, USA) was employed with size-selected, adaptor-ligated cDNA at 37°C for 15 min, and then at 95 °C for 5 min. Next, PCR was conducted with Universal PCR primers, Index (X) Primer, and Phusion High-Fidelity DNA polymerase. Using Agilent Bioanalyzer 2100 system to assess the library quality followed by PCR products purified (AMPure XP system). Clustering of the index-coded specimens was conducted using the TruSeq PE Cluster Kit v3-cBot-HS (Illumina) on a cBot Cluster Generation System. After clustering, the library preparation was sequenced on the Illumina platform and 150 bp paired-end reads were obtained.

Data analysis was performed with fastp v0.19.3 to filter the raw data by removing reads with adapters, removing paired reads with the N content in any sequencing read exceeding 10% of the base number of the reads, removing paired reads when the number of low-quality (Q ≤ 5) bases exceeded 50% of the bases in the reads. All clean reads were subjected to further analyses. We used featureCounts v1.6.2 to measure the FPKM values and gene alignment. DESeq2 v1.22.1 was employed for DEG analysis, and multiple testing was accounted for using the Benjamini & Hochberg method. The screening conditions for DEGs were False Discovery Rate < 0.05 and |log2Fold Change| ≥ 1. The enrichment analysis was conducted according to the hypergeometric test. For KEGG, the hypergeometric distribution test was conducted using the unit of pathway.

Table S1

Information on differential metabolites in water group compared with control group

| **Differential metabolites** | **Formula** | **VIP** | **Log2FC** | ***P*-vlaue** | **Trend** |
| --- | --- | --- | --- | --- | --- |
| POS |  |  |  |  |  |
| 2-Aminobenzamide | C7H8N2O | 1.8933 | 1.8132 | 0.0158 | up |
| L-Valine | C5H11NO2 | 1.8963 | 1.1681 | 0.0018 | up |
| Creatine | C4H9N3O2 | 1.7382 | 1.8574 | 0.0214 | up |
| 4,6-Diamino-5-formamidopyrimidine | C5H7N5O | 1.1931 | 1.0946 | 0.0367 | up |
| (2R)-2,3-Dihydroxypropanoic acid | C3H6O4 | 1.5602 | 2.1118 | 0.0027 | up |
| N-Carboxyethyl-gamma-aminobutyric acid | C7H13NO4 | 1.5818 | 1.4510 | 0.0218 | up |
| Myriocin | C21H39NO6 | 1.6317 | 2.2957 | 0.0396 | up |
| 3-aminobenzamide | C7H8N2O | 1.6983 | 1.0649 | 0.0045 | up |
| 1-Methylnicotinamide | C7H9N2O | 1.7884 | 1.0283 | 0.0072 | up |
| Sn-Glycero-3-Phosphocholine | C8H20NO6P | 1.6140 | 1.9901 | 0.0369 | up |
| Stearoyl-L-carnitine | C25H49NO4 | 1.6252 | 2.0068 | 0.0418 | up |
| 5'-Deoxy-5'-(Methylthio) Adenosine | C11H15N5O3S | 1.6228 | 2.0424 | 0.0324 | up |
| Carnitine C3:0 | C10H19NO4 | 1.7746 | 1.9098 | 0.0313 | up |
| D-Erythronic acid gamma-lactone | C4H6O4 | 1.6194 | 1.5267 | 0.0408 | up |
| Maltol | C6H6O3 | 1.7064 | 1.8831 | 0.0326 | up |
| N-Methylnicotinamide | C7H8N2O | 1.5259 | 1.1133 | 0.0356 | up |
| LPE(18:1/ 0:0) | C23H46NO7P | 1.8935 | -1.4102 | 0.0002 | down |
| LPE(0:0/20:4) | C28H50NO7P | 1.7629 | -1.5421 | 0.0030 | down |
| Ganoderic acid A | C30H44O7 | 1.6224 | -1.1006 | 0.0069 | down |
| Pantothenic acid | C9H17NO5 | 1.6212 | -1.3874 | 0.0064 | down |
| 3-Hydroxy-L-kynurenine | C10H12N2O4 | 1.7595 | -1.4384 | 0.0007 | down |
| 1-Palmitoyl-2-hydroxy-sn-glycero-3-phosphoethanolamine | C21H44NO7P | 1.9032 | -1.8717 | 0.0010 | down |
| Verapamil | C27H38N2O4 | 1.9609 | -1.2038 | 0.0002 | down |
| Chrysophanol | C15H10O4 | 1.3300 | -1.1359 | 0.0497 | down |
| Disopyramide | C21H29N3O | 1.7827 | -1.5627 | 0.0027 | down |
| LPC(18:1/0:0) | C26H53NO7P | 1.7394 | -1.3886 | 0.0025 | down |
| 5-Methoxyindole-3-acetic acid | C11H11NO3 | 1.6246 | -1.2221 | 0.0088 | down |
| N-Desmethyltapentadol | C13H21NO | 1.4760 | -1.3856 | 0.0409 | down |
| N-Acetylneuraminic Acid | C11H19NO9 | 1.2759 | -1.2069 | 0.0479 | down |
| 1-Pentadecanoyl-sn-glycero-3-phosphocholine | C23H48NO7P | 1.9198 | -1.4648 | 0.0002 | down |
| 3-Methylglutarylcarnitine | C13H23NO6 | 1.8375 | -1.1840 | 0.0039 | down |
| LPE(16:1/0:0) | C21H42NO7P | 1.9256 | -1.6454 | 0.0004 | down |
| LPC(16:1/0:0) | C24H49NO7P | 1.7695 | -1.4944 | 0.0023 | down |
| His-Asn-Ala | C13H20N6O5 | 1.7091 | -2.6164 | 0.0066 | down |
| Glu-Leu-Arg | C17H32N6O6 | 1.3147 | -1.4323 | 0.0128 | down |
| (2S)-4-[[(2S,3S,4R,5R)-5-(6-aminopurin-9-yl)-3,4-dihydroxyoxolan-2-yl]methylsulfanyl]-2-azaniumylbutanoate | C14H20N6O5S | 1.5944 | -1.7494 | 0.0169 | down |
| NEG |  |  |  |  |  |
| Pentachlorophenol | C6HCl5O | 1.0441 | 4.5872 | 0.0350 | up |
| UDP-N-acetyl-alpha-D-galactosamine | C17H27N3O17P2 | 1.3051 | 2.5735 | 0.0350 | up |
| Uridine-5'-monophosphate | C9H13N2O9P | 1.1401 | 2.5541 | 0.0368 | up |
| Adenosine | C10H13N5O4 | 1.2446 | 1.2083 | 0.0405 | up |
| L-Glutamic Acid | C5H9NO4 | 1.6422 | 1.8082 | 0.0060 | up |
| L-Aspartic Acid | C4H7NO4 | 1.7180 | 1.7555 | 0.0087 | up |
| 9-Hydroperoxy-10E,12Z-octadecadienoic acid | C18H32O4 | 1.3647 | 1.0034 | 0.0127 | up |
| L-Erythrulose | C4H8O4 | 1.3920 | 1.5205 | 0.0079 | up |
| Gluconic Acid | C6H12O7 | 1.6078 | 1.4629 | 0.0019 | up |
| O-Acetyl-L-serine | C5H9NO4 | 1.1884 | 1.9077 | 0.0192 | up |
| Phenaceturic acid | C10H11NO3 | 1.2293 | 1.3119 | 0.0312 | up |
| UDP-D-galactose | C15H24N2O17P2 | 1.5478 | 3.2057 | 0.0174 | up |
| UDP-glucose | C15H24N2O17P2 | 1.4836 | 4.1635 | 0.0305 | up |
| Inosine 5'-monophosphate | C10H13N4O8P | 1.0576 | 2.6928 | 0.0386 | up |
| 2-Aminoethanesulfonic acid | C2H7NO3S | 1.5905 | 2.0823 | 0.0130 | up |
| Aminomalonic Acid | C3H5NO4 | 1.3770 | 3.0319 | 0.0471 | up |
| [5-[[[5-carbamoyl-3-[3,4-dihydroxy-5-(phosphonooxymethyl)oxolan-2-yl]imidazol-4-yl]amino]methylideneamino]-3,4-dihydroxyoxolan-2-yl]methyl dihydrogen phosphate | C15H25N5O15P2 | 1.2784 | 2.6701 | 0.0443 | up |
| Uridine diphosphategalactose | C15H24N2O17P2 | 1.4735 | 2.5403 | 0.0141 | up |
| (2S)-2-azaniumylpropanoate | C3H7NO2 | 1.7298 | 2.6545 | 0.0021 | up |
| Stypandrol | C26H22O6 | 1.2196 | -1.6793 | 0.0117 | down |
| (±)8-HETE | C20H32O3 | 1.4820 | -2.1396 | 0.0024 | down |
| 9(S),12(S),13(S)-TriHOME | C18H34O5 | 1.6918 | -4.1983 | 0.0027 | down |
| (-)-Epigallocatechin | C15H14O7 | 1.3891 | -1.1169 | 0.0038 | down |
| (±)17-HDHA | C22H32O3 | 1.3783 | -1.0045 | 0.0131 | down |
| Quinolinic acid | C7H5NO4 | 1.7651 | -2.1047 | 4.0628E-05 | down |
| Dibutyl phthalate | C16H22O4 | 1.2997 | -2.3731 | 0.0281 | down |
| Bromobenzene-3,4-dihydrodiol | C6H7BrO2 | 1.4632 | -1.2022 | 0.0008 | down |
| (±)12-HEPE | C20H30O3 | 1.4962 | -1.8175 | 0.0010 | down |
| Quinalizarin | C14H8O6 | 1.3781 | -1.4902 | 0.0068 | down |
| 2,3-Dimercaptopropane-1-sulfonic acid | C3H8O3S3 | 1.4499 | -1.1890 | 0.0011 | down |
| 9(S)-HODE | C18H32O3 | 1.4905 | -1.9440 | 0.0014 | down |
| Ile-Thr | C10H20N2O4 | 1.4724 | -1.2310 | 0.0060 | down |
| 2-hydroxyquinoline | C9H7NO | 1.7191 | -1.1366 | 0.0007 | down |
| Uridine | C9H12N2O6 | 1.4146 | -1.6570 | 0.0046 | down |
| L-Tryptophan | C11H12N2O2 | 1.3740 | -1.1896 | 0.0097 | down |
| Cytochalasin H | C30H39NO5 | 1.6657 | -1.4065 | 0.0002 | down |
| Vasicinol | C11H12N2O2 | 1.2113 | -1.1669 | 0.0062 | down |
| 16-Hydroxyhexadecanoic acid | C16H32O3 | 1.3794 | -1.6491 | 0.0019 | down |
| L-Isoisoleucine | C6H13NO2 | 1.3318 | -1.5725 | 0.0131 | down |
| Nabumetone | C15H16O2 | 1.3032 | -1.2502 | 0.0172 | down |
| Metamitron | C10H10N4O | 1.3832 | -1.6203 | 0.0042 | down |
| Palmitoleic acid | C16H30O2 | 1.5258 | -1.9474 | 0.0017 | down |
| Galactinol | C12H22O11 | 1.3933 | -3.0178 | 0.0209 | down |
| Myricanone | C21H24O5 | 1.3938 | -1.6762 | 0.0044 | down |
| Ethynodiol diacetate | C24H32O4 | 1.4664 | -1.0055 | 0.0014 | down |
| 2-[4-[2-[4-cyclohexylbutyl(cyclohexylcarbamoyl)amino]ethyl]phenyl]sulfanyl-2-methylpropanoic acid | C29H46N2O3S | 1.5598 | -1.5197 | 0.0004 | down |
| 12,13-DiHOME | C18H34O4 | 1.4502 | -1.2917 | 0.0051 | down |
| 3,7-Dihydroxy-12-oxocholanoic acid | C24H38O5 | 1.0942 | -1.6432 | 0.0376 | down |
| Argininosuccinic acid | C10H18N4O6 | 1.3907 | -1.8139 | 0.0029 | down |
| 3-Oxocholic acid | C24H38O5 | 1.0850 | -1.8590 | 0.0473 | down |
| Psychosine | C24H47NO7 | 1.4410 | -1.1861 | 0.0039 | down |
| FFA(20:3) | C20H34O2 | 1.4612 | -1.4586 | 0.0017 | down |
| 1-Oleoyl-sn-glycero-3-phosphocholine | C26H52NO7P | 1.4717 | -1.2345 | 0.0024 | down |
| 8,9-DHET | C20H34O4 | 1.3583 | -1.3081 | 0.0066 | down |
| 6-(2,6-Dichlorophenyl)-2-{[3-(hydroxymethyl)phenyl]amino}-8-methylpyrido[2,3-D]pyrimidin-7(8H)-one | C21H16Cl2N4O2 | 1.3763 | -1.4384 | 0.0090 | down |
| Cortexolone | C21H30O4 | 1.4393 | -1.9200 | 0.0041 | down |
| (Rs)-Mevalonic Acid | C6H12O4 | 1.3690 | -1.2981 | 0.0021 | down |
| Mevalonic acid 5-pyrophosphate | C6H14O10P2 | 1.3834 | -1.5201 | 0.0061 | down |
| Narirutin | C27H32O14 | 1.4996 | -3.3074 | 0.0116 | down |
| D-Calcium Pantothenate | C18H32CaN2O10 | 1.3546 | -1.5032 | 0.0103 | down |
| Pentosidine | C17H26N6O4 | 1.4607 | -1.0443 | 0.0014 | down |
| Azelaic acid | C9H16O4 | 1.7336 | -1.2359 | 0.0028 | down |
| Linoleic acid | C18H32O2 | 1.3209 | -1.3642 | 0.0113 | down |
| 4-Hydroxy-3-methoxybenzaldehyde | C8H8O3 | 1.4298 | -2.8583 | 0.0314 | down |
| Carbocysteine | C5H9NO4S | 1.2244 | -1.995 | 0.0217 | down |
| 2-Aminoethyl dihydrogen phosphate | C2H8NO4P | 1.4405 | -1.7480 | 0.0016 | down |
| 13(R)-HODE | C18H32O3 | 1.4244 | -1.8357 | 0.0086 | down |
| hydron | H+ | 1.5451 | -1.7761 | 0.0161 | down |
| N-lactoyl-phenylalanine | C12H15NO4 | 1.4681 | -1.7908 | 0.0014 | down |
| Macrocarpal I | C28H42O7 | 1.2014 | -1.5149 | 0.0382 | down |
| LysoPA(18:1(9Z)/0:0) | C21H41O7P | 1.6429 | -1.5807 | 0.0010 | down |
| Lys-Phe-Leu-Glu | C26H41N5O7 | 1.6392 | -1.1635 | 0.0004 | down |
| Leu-Gln-Asn-Arg | C21H39N9O7 | 1.5209 | -1.3637 | 0.0017 | down |
| Leu-Ala-Thr-Lys | C19H37N5O6 | 1.5943 | -1.6526 | 0.0008 | down |
| LPE(18:1/0:0) | C23H46NO7P | 1.5664 | -1.5207 | 0.0004 | down |
| LPE(18:0/0:0) | C23H48NO7P | 1.6287 | -1.5857 | 0.0004 | down |
| LPE(16:0/0:0) | C21H44NO7P | 1.6135 | -2.3460 | 0.0011 | down |
| LPE(0:0/18:0) | C23H48NO7P | 1.6110 | -1.8020 | 0.0004 | down |
| LPE(0:0/16:0) | C21H44NO7P | 1.6637 | -1.2093 | 0.0001 | down |
| Ile-Ser-Arg-Lys | C21H42N8O6 | 1.1137 | -1.2377 | 0.0324 | down |
| Glutathione Reducedform | C10H17N3O6S | 1.1950 | -1.2138 | 0.0344 | down |
| D-Urobilinogen | C33H42N4O6 | 1.5537 | -1.1070 | 0.0008 | down |
| Asn-Arg-Phe-Lys | C25H41N9O6 | 1.6196 | -1.4295 | 0.0006 | down |
| Arachidonic Acid | C20H32O2 | 1.3244 | -1.3796 | 0.0080 | down |
| 9,10-EpOME | C18H32O3 | 1.7659 | -2.5883 | 0.0008 | down |
| 7-Hydroxy-6-methyl-8-ribityl lumazine | C12H16N4O7 | 1.1387 | -1.3432 | 0.0331 | down |
| 6-Deoxy-6-sulfo-D-fructose 1-phosphate | C6H13O11PS | 1.3876 | -1.5143 | 0.0055 | down |
| 5(S),6(R)-11-trans DiHETE | C20H32O4 | 1.3457 | -1.0261 | 0.0105 | down |
| 4-amino-6,7-dihydro-5H-cyclopenta[b]pyridin-2-yl 4-methylbenzenesulfonate | C15H16N2O3S | 1.2528 | -1.0507 | 0.0283 | down |
| 4-CDP-2-C-methyl-D-erythritol | C14H25N3O14P2 | 1.3302 | -1.3516 | 0.0156 | down |
| 16-Oxo-palmitate | C16H30O3 | 1.4576 | -1.7376 | 0.0069 | down |
| 12(13)-EpOME | C18H32O3 | 1.3839 | -2.0239 | 0.0006 | down |
| 11,12-dihydroxy-5Z,8Z,14Z-eicosatrienoic acid | C20H34O4 | 1.4358 | -2.9287 | 0.0178 | down |
| 1-O-Hexadecyl-2-deoxy-2-thio-S-acetyl-sn-glyceryl-3-phosphorylcholine | C26H54NO6PS | 1.5777 | -1.3086 | 0.0004 | down |
| (2R,3R,4S,5S,6R)-6-[[(2S,3R,4S,5R,6R)-3,4,5-trihydroxy-6-(hydroxymethyl)oxan-2-yl]oxymethyl]oxane-2,3,4,5-tetrol | C12H22O11 | 1.3600 | -2.3150 | 0.0206 | down |
| (2R)-2-methyl-2-[(2S,3R,4S,5S,6R)-3,4,5-trihydroxy-6-(hydroxymethyl)oxan-2-yl]oxybutanenitrile | C11H19NO6 | 1.2425 | -1.4407 | 0.0277 | down |


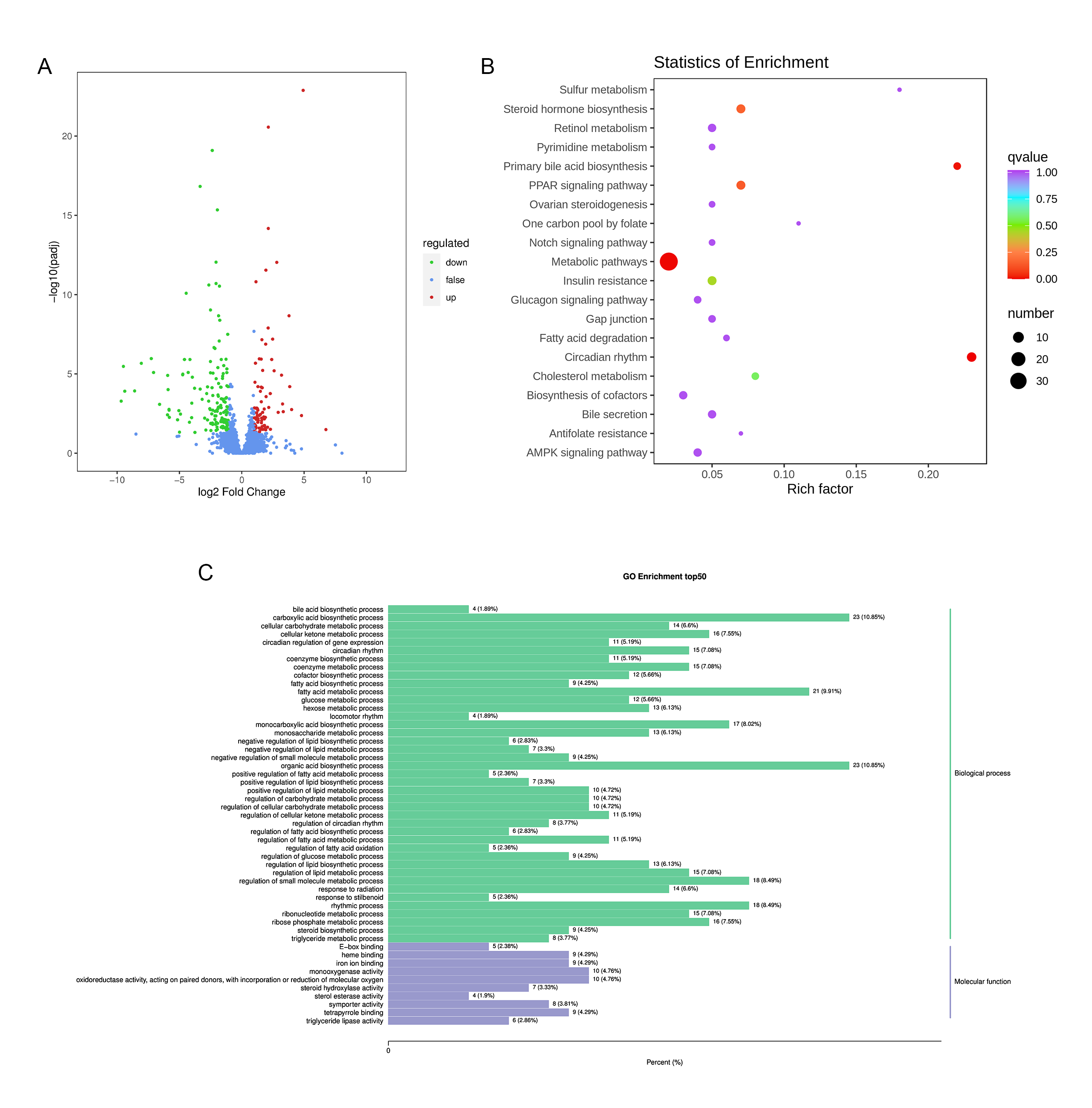


Fig S1 Effects of C-cell water on liver transcriptome. A. The volcano plot of differential genes between C-cell water and control groups. B. Enriched KEGG pathways based on significantly different genes of C-cell water *vs* ultrapure water. C. Enriched KEGG pathways based on significantly different genes of C-cell water *vs* ultrapure water.
